# Ischemia/reperfusion injury in acute human and experimental stroke: focus on thrombo-inflammatory mechanisms and treatments

**DOI:** 10.1186/s42466-024-00355-y

**Published:** 2024-11-25

**Authors:** Guido Stoll, Bernhard Nieswandt, Michael K. Schuhmann

**Affiliations:** 1 Institute of Experimental Biomedicine I, University Hospital Wurzburg, Josef-Schneider-Str. 2, 97080 Wurzburg, Germany; 2 Rudolf Virchow Center, Center for Integrative and Translational Biomaging, University of Wurzburg, Josef-Schneider-Str. 2, 97080 Wurzburg, Germany; 3 Department of Neurology, University Hospital Wurzburg, Josef-Schneider-Str. 11, 97080 Wurzburg, Germany

**Keywords:** Ischemic stroke, Ischemia/reperfusion injury, Thrombo-inflammation, Glycoprotein receptor VI, Toll like receptor 4, Platelet-leukocyte interactions, Pial blood sampling, Clinical trials

## Abstract

**Background:**

Despite high recanalization rates of > 90% after endovascular thrombectomy (EVT) clinical outcome in around 50% of treated acute ischemic stroke (AIS) patients is still poor. Novel treatments augmenting the beneficial effects of recanalization are eagerly awaited, but this requires mechanistic insights to explain and overcome futile recanalization.

**Main body:**

At least two mechanisms contribute to futile recanalization after cerebral large vessel occlusions (LVO): (i) the no reflow phenomenon as evidenced by randomly distributed areas without return of blood flow despite reperfusion of large cerebral arteries, and (ii) ischemia/reperfusion (I/R) injury, the paradoxically harmful aspect of blood flow return in transiently ischemic organs. There is accumulating evidence from experimental stroke models that platelets and leukocytes interact and partly obstruct the microvasculature under LVO, and that platelet-driven inflammation (designated thrombo-inflammation) extends into the reperfusion phase and causes I/R injury. Blocking of platelet glycoprotein receptors (GP) Ib and GPVI ameliorated inflammation and I/R injury providing novel therapeutic options. Recently, MRI studies confirmed a significant, up to 40% infarct expansion after recanalization in AIS thereby proofing the existance of I/R injury in the human brain. Moreover, analysis of minute samples of ischemic arterial blood aspirated directly from the pial cerebral collateral circulation under LVO during the routine EVT procedure confirmed platelet activation and platelet-driven leukocyte accumulation in AIS and, thus, the principal transferability of pathophysiological stroke mechanisms from rodents to man. Two recently published clinical phase 1b/2a trials targeted (thrombo-) inflammation in AIS: The ACTIMIS trial targeting platelet GPVI by glenzocimab provided encouraging safety signals in AIS similar to the ApTOLL trial targeting toll-like receptor 4, a central receptor guiding stroke-induced innate immunity. However, both studies were not powered to show clinical efficacy.

**Conclusions:**

The fact that the significance of I/R injury in AIS has recently been formally established and given the decisive role of platelet-leukocytes interactions herein, new avenues for adjunct stroke treatments emerge. Adjusted study designs to increase the probability of success are of outmost importance and we look forward from what can be learned from the so far unpublished, presumbably negative ACTISAFE and MOST trials.

## Background

Stroke is the second leading cause of death and the third frequent cause of permanent disability worldwide. Despite more effective prevention measures absolute stroke numbers constantly increase due to the aging population and an increasing life expectancy in developing countries [53]. In acute ischemic stroke (AIS) the most effective therapeutic measure has been rapid reconstitution of blood flow by recanalization [30]. Recanalization can be achieved by pharmacological thrombolysis induced by systemic application of tissue plasminogen activator (t-PA) or more recently tenecteplase with a broad indication not depending on the demonstration of an occluded major cerebral artery [4, 13] and/or by endovascular thrombectomy (EVT) which relies on the presence of a large vessel occlusion (LVO) amenable to mechanical thrombus retrieval [30]. Thrombolysis and EVT have been constantly refined and the time windows extended. Full recanalization rates by EVT (indicated by modified treatment in cerebral ischemia (mTICI) scale grades ≥ 2b [73]) reach about 90%, but even after technically successful EVT, the individual prognosis of LVO-stroke remains poor with only about 35–55% favorable outcome and 14–28% mortality at 3 months [47, 71]. Recanalization obviously is a prerequisite for a better functional outcome, but a large group of treated patients experience futile recanalization. Thus, there is an urgent need to develop add-on treatments beyond recanalization [45, 65, 69]. Possible reasons for futile recanalization have for long been identified in experimental stroke models and, among others, two principal mechanisms emerged: the no reflow phenomenon [5] and ischemia/reperfusion (I/R) injury [21, 50] which are both partly driven by platelet-leukocyte interactions and interrelated [64]. In 2011, we coined the term “thrombo-inflammation” to designate platelet-driven inflammation which is of fundamental pathophysiological importance particularly in AIS (see below) [49, 64]. In the following we will (i) briefly describe how the concept of thrombo-inflammation as key feature of I/R injury emerged from experimental stroke models, (ii) review recent evidence for infarct progression despite full recanalization (I/R injury) in human AIS, (iii) summarize evidence for intravascular platelet-driven inflammation commencing under LVO in human stroke patients, and (iv) discuss the results of recently completed, inflammation-related clinical trials.

## Main text

### The impact of thrombo-inflammation on the no reflow phenomenon and ischemia/reperfusion injury in experimental stroke

#### No reflow phenomenon

**Fig. 1:**
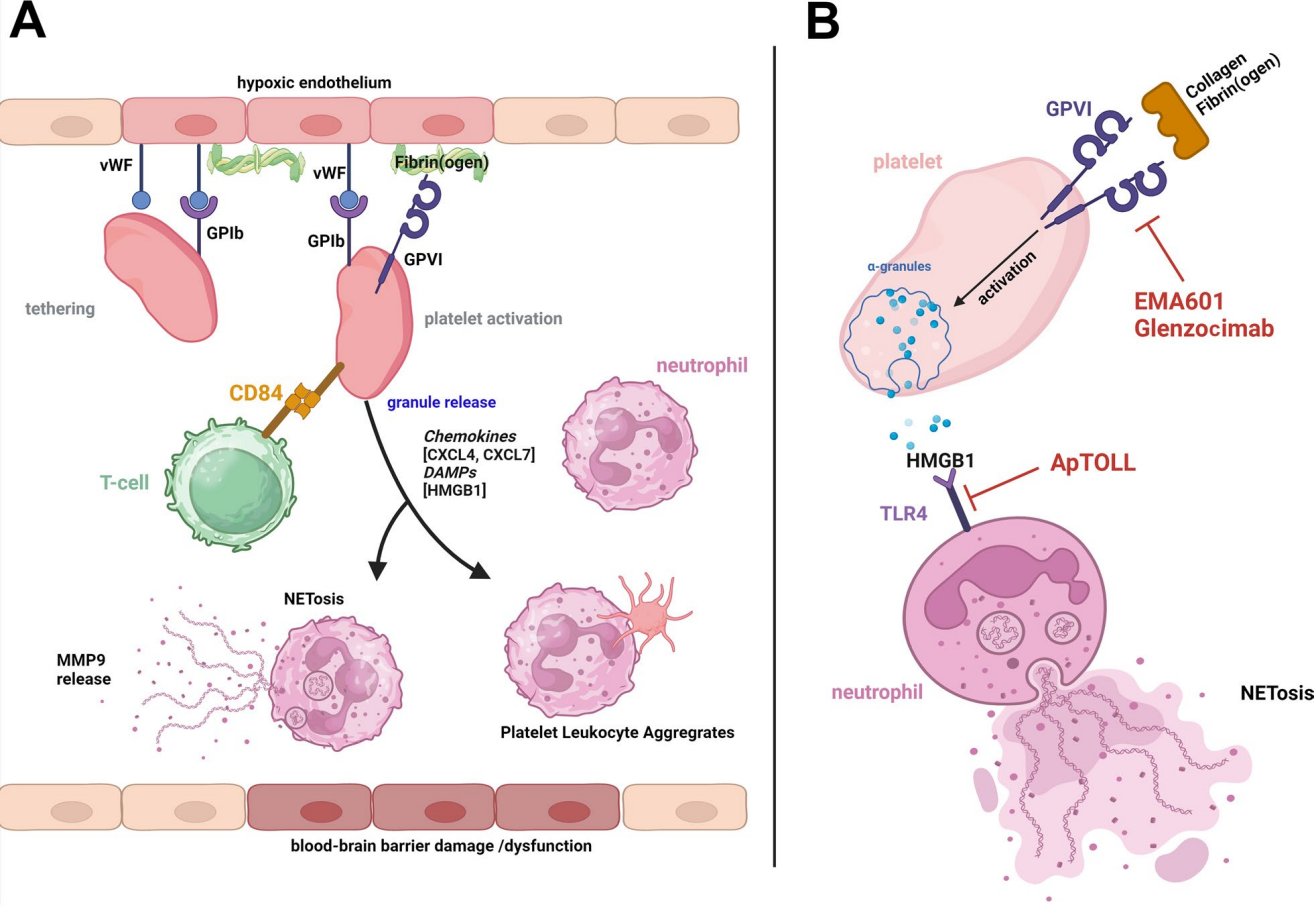
Signaling pathways of thrombo-inflammation in acute ischemic stroke as novel targets for treatment. **A** illustrates thrombo-inflammation as it denotes the complex interaction between platelets and neutrophils/T-cells leading to tissue injury without requiring thrombus formation. During cerebral artery occlusion as well as in the reperfusion phase platelets tether by binding of GPIb to von Willebrand Factor (vWF) exposed on the hypoxic endothelial surface, and in a second step, become activated via GPVI binding to fibrin(ogen), collagen and/or other unknown ligands. This leads to platelet release of α-granules, which contain the chemokines CXCL4, CXCL7 as well as damage-associated molecular patterns (DAMPs) such as HMGB1 among others. CXCL7 is a potent chemoattractant for neutrophils, and CXCL4 can induce granule release from neutrophils, including the secretion of MMP9. HMGB1 is a potent inducer of NET formation. MMP9 and NETosis can cause disruption of the blood–brain barrier and perivascular tissue damage. In addition, T-cells interact with platelets via CD84, a homophilic cell adhesion molecule, and by so far unknown effector mechanisms aggravate infarct progression. **B** highlights targets of emerging anti-thrombo-inflammatory treatments: EMA601 and glenzocimab block GPVI signaling and/or binding while ApTOLL is a TLR4 antagonist. TLR4 is strongly expressed on neutrophils and serves as a major receptor for DAMPs, such as HMGB1. TLR4 signaling can induce NETosis in neutrophils. Figure created with BioRender.com

The observation that blood does not flow despite recanalization has been termed no reflow phenomenon [5]. Ames et al. in 1968 demonstrated that relief of obstruction following prolonged cerebral ischemia in rabbits did not restore normal blood flow as indicated by randomly distributed hypoperfused pale brain regions when colloid carbon was systemically applied [5]. It has been proposed that during ischemia the luminal surface of endothelium within the microvasculature eventually converts from an anticoagulant to a procoagulant membrane [31]. In support of this view deposits of radioactively labeled platelets were detectable in ischemic basal ganglia very early during reperfusion in a primate model of transient middle cerebral artery occlusion (tMCAO) [16]. Electron microscopic examination of the microvasculature within the ischemic region further demonstrated aggregates of degranulated platelets together with fibrin and leukocytes and provided direct evidence that platelet activation occurs in the ischemic zone [15, 51]. Recently, the presence of leukocytes and platelet-leukocyte aggregates partly, but not completely obstructing microvessels has been confirmed by modern imaging modalities [19]. In support of this concept, we could further show that platelets tether to the vessel wall via binding of the platelet receptor glycoprotein (GP) Ib to von Willebrand factor [57], and then become activated by GPVI/collagen and/or GPVI/fibrin(ogen) interactions during cerebral ischemia in a rodent model under MCAO already before recanalization (Fig. 1A) [8]. Functionally, blocking of GPIb [57] or depletion of GPVI [8] attenuated infarct growth under LVO before recanalization and reduced the intravascular accumulation of leukocytes. Thus, intravascular platelet-leukocyte interactions emerge already under LVO and may partly explain the no reflow phenomenon upon recanalization [66]. Further components potentially involved in the no reflow phenomenon have been reviewed in detail recently elsewhere [63].

#### Ischemia/reperfusion (I/R) injury

In experimental animals cerebral infarcts further grow and maturate within the following 24 h despite full recanalization as shown in the widely used transient MCAO model [9, 28]. In general, the paradoxically harmful aspect of blood flow return in transiently ischemic organs has been termed I/R injury and applies to many other organs such as the heart, kidney, lung and liver [21, 31]. While many additional factors such as oxidative stress, protein synthesis suppression, apoptosis, disruption of the neurovascular unit may contribute to I/R injury as discussed in detail elsewhere [7, 50], we here focus on platelet activation and leukocyte infiltration.

In mice, a short MCA occlusion time of only one hour finally leads to a complete infarction of the MCA territory developing during reperfusion [9]. An elegant study employing light sheet microscopy by Göb et al. [28] could show that despite recanalization induced by retracting the MCA occluding filament at one hour, complete MCA infarcts gradually developed in mice within the following 8 h, but, surprisingly, at that time no significant thrombus formation occurred within the ischemic hemisphere. Intravascular thrombi were regularly detectable only later at 23 h after transient MCAO long after infarct development. This strongly indicates that infarct growth after recanalization (I/R injury) cannot primarily be driven by re-thrombosis of re-perfused cerebral macro- and microvessels, but is at least partly due to a detrimental interaction between platelets, immune and endothelial cells designated thrombo-inflammation [49, 64]. In contrast to firm thrombus formation, in which GPIIb/IIIa-mediated platelet aggregation is mandatory, platelet GPIb and GPVI in I/R injury guide inflammation after cerebral ischemia without involvement of GPIIb/IIIa signaling [37]. Blocking of platelet GPIb or GPVI not only attenuated infarct growth under LVO before recanalization as described above, but, moreover, largely protected mice from cerebral I/R injury after recanalization and thereby improved clinical outcomes [37, 58, 60]. This indicates a continuum of platelet activation amenable to treatment starting already during LVO, but continuing despite recanalization and causing I/R injury. Importantly, no bleeding complications were seen upon GPIb or GPVI blockade [37, 42, 60] in contrast to the futile treatment with anti-GPIIb/IIIa Fab fragments or humanized antibodies leading to severe intracranial hemorrhages (ICH) both in experimental [37] and clinical [2] stroke.

Functionally, platelets during I/R injury primarily drive inflammation (e.g. T-cell and neutrophil responses) rather than (re-) thrombus formation (Fig. 1A). This notion is further supported by stroke experiments in immune-deficient mice. *Rag1*^*−/−*^ mice lacking T- and B-cells are protected from I/R injury after transient MCAO [38, 72]. Upon adoptive transfer of T-, but not B-cells, *Rag1*^*−/−*^ mice are fully susceptible to I/R injury again. Importantly, the detrimental effect of T-cells does not require recognition of a specific antigen, but depends on the presence of platelets [36]. Recently, CD84, a homophilic cell adhesion molecule could be identified as the mechanistic link between detrimental effects of T-cells and platelets in stroke (Fig. 1A) [61]. During human stroke, CD84 is shed from the surface of platelets and, in vitro, soluble CD84 increases T cell mobility via homophilic CD84-CD84 interactions.

Neutrophils are the predominant leukocytes rapidly accumulating within the intravascular cerebral compartment and with a delay > 24 h infiltrate the ischemic brain parenchyma in experimental and human AIS [22, 41, 66, 70]. Platelets can activate neutrophils in particular upon platelet-neutrophil aggregate formation. Activated neutrophils expel neutrophil extracellular traps (NET) (Fig. 1A,B) which represent extracellular DNA lattices trapping pathogens during host defense to combat infections [62], but also platelets and coagulation factors with ensuing thrombus formation. NET formation occurs in experimental and human stroke and contributes to ischemic lesion development [17, 68]. Thereby, platelet derived high-mobility group box 1 (HMGB1) is instrumental, underpinning the important role of platelet-neutrophil interactions in AIS. Accordingly, treatment with a neonatal NET inhibitory factor reduced I/R injury and ameliorated clinical outcome in a mouse tMCAO model [17].

The expression of the Toll like receptor (TLR) 4 on neutrophils [18] provides a possible link between platelet HMGB1 release and neutrophil activation including NET formation (Fig. 1B). TLR4 is critically involved in the induction of innate immune responses and usually activated by bacteria-released lipopolysaccharide during infections, but also damage-associated molecular patterns (DAMPs) such as HMGB1 [6]. Local HMGB1 concentrations are significantly increased in experimental and clinical stroke [17, 59]. TLR4 deficient mice are protected from I/R injury in stroke [12]. Since TLR4 is expressed on many cell types beyond leukocytes including neurons interpretation of the beneficial result is difficult, but there is one intriguing observation: In *Tlr4*^*−/−*^ mice, paradoxically higher numbers of neutrophils had infiltrated the ischemic brain 48 h after stroke compared with WT mice [26]. However, TLR4 deficiency increased the levels of alternatively polarized N2 neutrophils with neuroprotective features in ischemic lesions. Thus, it appears that TLR4 signaling fosters detrimental N1 neutrophil responses, while TLR4 deficiency supports polarization towards a beneficial N2 phenotype [26]. The disparity of neutrophil populations during different stages of infarct development and reorganization may explain the controversial net effects of gross neutrophil depletion on outcomes described in experimental stroke models and the treatment failures when targeting neutrophil migration in human AIS [35, 46, 56].

Although the effector pathways of these complex T-cell and neutrophil responses in AIS await further elucidation, there is accumulating evidence that platelets guide this inflammation by engagement of GPIb and GPVI receptors and granule release (including HMGB1) beyond thrombus formation in experimental and clinical stroke which provides novel therapeutic perspectives (see below) (Fig. 1A,B).

### Evidence for cerebral infarct expansion despite recanalization (I/R injury) in human stroke

At present it is difficult to assess and quantify the extent of the no reflow phenomenon post recanalization in human stroke [63]. In contrast, the existence and significant impact of I/R injury has recently been proven [33, 54, 55]. Given the major and well documented impact of I/R injury on progressive organ dysfunction in the reperfused heart, lung, liver and kidney [21] it is surprising that cerebral I/R injury has long been neglected/questionend [27]. The establishment of EVT in 2015/2016 as a standard treatment in AIS upon LVO profoundly changed the scientific scene and enabled sequential magnetic resonance imaging (MRI) studies on stroke progression appropriately controlled for the degree of recanalization achieved [30]. EVT requires routine angiography during the procedure and thereby enables precise grading of individual recanalization success e.g. by the mTICI score. Three recent MRI studies reported the development of infarct expansion in AIS patients measured shortly before or after EVT and during follow-up covering the reperfusion phase.

Sah and colleagues [55] compared lesion volumes on MR-diffusion weighted images (DWI) obtained at 5 h (initial posttreatment) and 24 h (follow-up) after EVT in 33 AIS patients and described a measurable growth of the DWI lesion even in patients with the best recanalization on digital subtraction angiography post thrombectomy of mTICI-3. These findings were confirmed and extended by Rex and colleagues [54] in a larger cohort showing a lesion expansion on DWI-MRI on average of 39% in 151 patients between 2 and 24 h after EVT which was seen in 88% of patients. Interestingly, relative lesion expansion was consistent across all TICI categories, but greater absolute lesion expansion as typically seen in patients with large infarctions before EVT was associated with worse outcome. Finally, Hernandez-Perez and colleagues [33] in a prospective study performed DWI-MRI on arrival at the hospital (pre-EVT), ≤ 2 h after EVT (post-EVT) and on day 5 in 98 AIS patients. For the whole cohort median DWI volumes pre-EVT, post-EVT, and at 5 days were 12, 20 and 25 cc, respectively, indicating infarct grow before recanalization under LVO as well as after recanalization even in patients who achieved mTICI 3. The extent of late infarct growth in the available 77 patients was larger in those with higher NIHSS scores at baseline. Collectively, these studies showed a substantial increase in infarct volumes up to 40% despite successful recanalization in human AIS which, in principle, has been predicted from experimental stroke models. At present, it is unclear which mechanisms drive secondary infarct progression after recanalization in human stroke, but it is likely that similar to rodent stroke I/R injury involving thrombo-inflammation is a key element [50, 64]

### Evidence for leukocyte accumulation and local platelet activation in pial blood samples in human stroke

From the above mentioned studies on I/R injury in human stroke the question arises whether the mechanistic findings in experimental stroke also apply to humans. Obviously it is not possible to analyse brain tissue directly at ultra-early time points in AIS patients, but EVT opens a diagnostic window. It is possible to acquire minute samples of ischemic arterial blood aspirated directly from the pial cerebral collateral circulation by navigating a microcatheter through the embolic occlusive lesion immediately before EVT [25, 41]. As control a second sample is taken at the level of the internal carotid artery under physiological non-occlusive flow conditions. Comparing platelet responses and leukocyte counts between these paired individual samples allows the assessment of intravascular thrombo-inflammation within the secluded ischemic brain provisionally nourished by pial collateral blood flow. As main finding we detected a substantial increase in the number of neutrophils in the pial blood samples indicating an accumulating local inflammatory response [41, 74]. Moreover, there were significantly increased concentrations of platelet-derived neutrophil-activating chemokine CXC motif ligand (CXCL) 4 (platelet factor 4) and CXCL7 (neutrophil-activating 2 peptide) (Fig. 1A) [40]. While CXCL7 is a potent chemoattractant for neutrophils, CXCL4 induces neutrophil degranulation and increases their secretion of matrix metalloproteinase (MMP) 9 [10]. Interestingly, we also found increased local MMP9 plasma concentrations and an enhanced intracellular MMP9 expression within neutrophils by immunofluorescence [39]. MMP9 levels were predictive for the risk of major ICH and poor outcome before EVT, providing evidence for the validity of ultra-early local stroke biomarkers. On a mechanistic level, MMP9 plays a major role in the breakdown of the blood–brain barrier in experimental cerebral ischemia [67]. Thus, it is likely that the early thrombo-inflammatory response commencing under LVO and extending into the reperfusion phase as evidenced in experimental stroke significantly contributes to additional brain damage beyond ischemia/hypoxia.

As mentioned above, platelets are an important source of HMGB1 [17], one of the most prevalent DAMPs which trigger sterile inflammation by activation of inflammasome pathways [29]. In pial occlusive intravascular samples local HMGB1 concentrations were increased by more than 30% in addition to another DAMP, calprotectin (S100A8/A9) which is mainly released by leukocytes [59]. Moreover, systemic HMGB1 levels rapidly increased within 24 h in venous blood samples and independently predicted long term clinical outcome [34].

Taken together these data suggest a vicious cycle of platelet-leukocyte interactions in hyper-acute stroke patients in which local DAMP and chemokine/cytokine release by platelets (e.g. HMGB1, CXCL4, CXCL7) and leukocytes (e.g. S100A8/A9, MMP9) entertain neuroinflammation directly within the vascular compartment and contribute to ischemic brain damage (Fig. 1A). Given that pharmaceutical substances such as t-PA reach the ischemic penumbra via pial collaterals in significant amounts under LVO [23], modifying this response may be feasible and promising. Results of recently completed trials targeting inflammation and/or secondary thrombus formation in AIS patients in combination with EVT and/or thrombolysis are summarized below.

### Recent clinical trials targeting I/R injury in acute ischemic stroke

The Acute Ischemic Stroke Interventional phase 1b/2a Trial (ACTIMS) targeted platelet GPVI by applying glenzocimab, a Fab fragment against human GPVI (Fig. 1B), within three hours after AIS onset in addition to alteplase with or without EVT [43]. After testing several concentrations in a phase 1b safety analysis, a dose of 1000 mg was selected for the phase 2a involving 54 patients receiving glenzocimab versus 52 patient placebo. In phase 2a the most frequent treatment-related overall adverse event was non-symptomatic hemorrhagic transformation occurring in 31% of patients treated with glenzocimab, and even at a higher percentage of 50% in the placebo group. No patient developed symptomatic ICH in the treatment group versus 10% in the placebo group. All-cause deaths were lower with glenzocimab (7% of patients) than in the placebo arm (21%). However, the highest dose of 1000 mg glenzocimab did not affect functional autonomy and mortality at 90 days, but the trial was underpowered to assess efficacy. Results of the ensuing clinical phase III trial (ACTISAVE) recruiting larger patient numbers and adequately powered were presented at the ESOC congress in Basel on May 15, 2024. Overall, the primary endpoint of clinical efficacy was not reached according to a press release by ACTICOR Biotech (Acticor [1]). Since publication of the final results is still pending, at present it is not possible to draw firm conclusions. However, the relatively low affinity of glenzocimab for GPVI as revealed by a recent comparative in-vitro analysis [48] requiring high doses for full blockade of the GPVI receptor may have been an issue. Furthermore, the very short in vivo half-life of glenzocimab may have resulted in a loss of GPVI inhibition while thrombo-inflammation was still ongoing. Novel GPVI inhibitors with a > 50-fold potency compared to glenzocimab and a superior experimental pharmacodynamic profile (assessed in GPVI-humanised mice) are currently under preclinical development and hold the promise of a more efficient GPVI inhibition that is, importantly, not associated with an increased bleeding risk [44, 48]. Importantly, the ACTIMIS trial supports the notion from numerous experimental investigations in different organ systems such as brain and lung, that targeting GPVI is safe without bleeding complications [11, 37, 48]. In their discussion, Mazighi and colleagues concluded that targeting GPVI in AIS might have modulated thrombo-inflammation and the downstream microcirculation by a reperfusion effect [43].

The Multi-Arm Optimizing of Stroke Thrombolysis (MOST) trial took a conventional approach. Based on the assumption that infarct growth after recanalization is due to re-embolization and/or local re-thrombosis the MOST trial evaluated the efficacy of adjunctive thrombolysis with the blood thinner, argatroban, a thrombin inhibitor, and eptifibatide, a reversible GPIIb/IIIa antagonist [14]. The trial was halted early after the first 500 enrolled patients did not show improvement according to a press release [3]. Neither of the blood thinners improved disability based on the modified Rankin scale at 90 days after stroke onset. The detailed trial results have not yet been published limiting firm interpretation. However, the overall negative outcome appears in line with previous investigations in which blockade of platelet aggregation by GPIIb/IIIa antagonists failed both in the tMCAO model [37] and in a human phase III trial [2] mainly due to severe bleeding complications which have not been seen with inhibitors of platelet receptors dispensable for hemostasis, most notably GPVI antagonists [43, 48, 64]. Moreover, the MOST trial appears to further substantiate experimental evidence that infarct progression after recanalization is not primarily due to re-thrombosis of the microvasculature, but to complex platelet-leukocyte interactions and other processes not requiring clot formation as discussed above.

To modify inflammation-mediated infarct progression, the APRIL double blind, randomized, multicenter, placebo-controlled phase Ib/IIa trial targeted TLR4 by applying ApTOLL, a DNA aptamer (Fig. 1B) [32]. Aptamers are single-stranded oligonucleotides that recognize and selectively bind target molecules with high specificity and nanomolar affinity [20]. They can be chemically synthesized and are non-immunogenic. TLR4-binding DNA aptamers showed a protective effect against acute stroke in rodent MCAO models [24]. In the APRIL trial, after dose finding during phase 1b, two doses, 0.05 mg/kg and 0.2 mg/kg of ApTOLL or placebo were administered intravenously within 6 h of stroke onset in combination with EVT in AIS patients with an Alberta Stroke Program Early CT score of 6 to 10. The primary composite endpoint was safety encompassing the incidence of death, sICH, malignant stroke and recurrent stroke and was reached in 16 of 55 patients (29%) receiving placebo but only in 6 of 42 patients (14%) receiving ApTOLL 0.2 mg/kg. The APRIL trial was not powered to achieve definite results in regard to clinical efficacy, but the preliminary data suggest a reduced mortality and disability at 90 days compared to placebo in the group treated with 0.2 mg/kg ApTOLL [32] which awaits confirmation from larger trials.

## Conclusions

The technical success rate of EVT in LVO stroke has reached a ceiling effect of > 90% recanalization, but treatment effects are still modest/insufficient while leaving around 50% of eligible AIS patients with significant disability or death. Attempts to improve outcomes by concomitant anticoagulation and recanalization have largely failed, in particular due to excess bleeding complications [2]. Targeting platelet receptors dispensable for hemostasis, but guiding inflammation such as GPVI [44, 48] or key effector receptors of stroke-related inflammation such as TLR4 [32], among others, are worth being pursued clinically based on robust preclinical efficacy and clinical safety data. Adjusted study designs which exclude patients either with excellent outcomes after EVT or with large infarcts may increase the probability of success by enriching the study population which might benefit most from an adjunct treatment under investigation [52].

## Data Availability

Data sharing is not applicable to this article as no datasets were generated or analysed during the study.

## Abbreviations

ACTIMIS: Acute ischemic stroke interventional trial
AIS: Acute ischemic stroke
CXCL: Neutrophil-activating chemokine CXC motif ligand
DAMPs: Damage-associated molecular patterns
DWI: Diffusion weighted imaging
EVT: Endovascular thrombectomy
GP: Glycoprotein receptor
HMGB1: High-mobility group box 1 protein
ICH: Intracranial hemorrhage
I/R injury: Ischemia/reperfusion injury
MCAO: Middle cerebral artery occlusion
MMP9: Matrix metalloproteinase 9
MOST: Multi-arm optimizing of stroke thrombolysis trial
MRI: Magnetic resonance imaging
mTICI: Modified treatment in cerebral ischemia scale
NETs: Neutrophil extracellular traps
LVO: Large vessel occlusion
t-PA: Tissue plasminogen activator
TLR4: Toll-like receptor 4
vWF: Von Willebrand Factor
